# Real architecture For 3D Tissue (RAFT™) culture system improves viability and maintains insulin and glucagon production of mouse pancreatic islet cells

**DOI:** 10.1007/s10616-017-0067-6

**Published:** 2017-02-08

**Authors:** Gabor J. Szebeni, Zsuzsanna Tancos, Liliana Z. Feher, Robert Alfoldi, Julianna Kobolak, Andras Dinnyes, Laszlo G. Puskas

**Affiliations:** 1 Avidin Ltd, Also kikötő sor 11/D, Szeged, H6726 Hungary; 2 Biotalentum Ltd, Szent-Györgyi Albert 4, Gödöllő, H2100 Hungary

**Keywords:** 3D culture, RAFT™, Pancreas islet, Insulin

## Abstract

There is an unmet medical need for the improvement of pancreatic islet maintenance in culture. Due to restricted donor availability it is essential to ameliorate islet viability and graft engraftment. The aim of this study was to compare the standard tissue culture techniques with the advanced Real Architecture For 3D Tissue (RAFT™) culture system in terms of viability and hormone production. Here, we first report that islets embedded in RAFT™ collagen type I advanced tissue culture system maintain their tissue integrity better than in monolayer and suspension cultures. The Calcein violet assay and Annexin V/propidium-iodide staining show higher cell viability in the RAFT™ culture system. Quantitative real-time PCR data showed that RAFT™ increases insulin expression after 18 days in culture compared to traditional methods. Enhanced insulin and glucagon production was further verified by immunofluorescent staining in a time-course manner. These results indicate that RAFT™ tissue culture platform can be a promising tool to maintain pancreatic islet spheroid integrity and culture islets for downstream high throughput pharmacological studies ex vivo.

**Electronic supplementary material:**

The online version of this article (doi:10.1007/s10616-017-0067-6) contains supplementary material, which is available to authorized users.

## Introduction

*Diabetes mellitus* (DM) affects millions worldwide. The cure of these multiple disorders due to the complex pathomechanisms is still a harboring challenge. The understanding of the pathogenesis of both type I, type II and gestational diabetes needs further experimental models. Development of novel, ex vivo tissue culture techniques maintaining pancreatic islets is critical in order to better understand the disease group and support the basis for improved transplantation therapies. All types of diabetes share the burden of high blood glucose level (hyperglycemia) which without interventions can cause life-threatening severe symptoms including ketoacidosis, blindness, heart failure and stroke (van Belle et al. 2011). Almost 90–95% of adult patients suffer from type 2 DM (Lysy et al. 2016). Both genetic and life style elements (obesity, unhealthy diet, low physical activity, stress and smoking) can contribute to the establishment of type 2 DM where muscle and adipose tissues are resistant to insulin produced by intact β-cells (Tsasis et al. 2016). Gestational diabetes occurs in approximately 16% of pregnant woman and tend to progress to type 2 DM for 15–50% of the patients within 5 years but in most of the cases can elapse by physical activity and dietary change (Gilinsky et al. 2015). Diabetes has a heterogeneous secondary form developed by various reasons e.g. malignancy, pancreatitis or medications accounting for a minority of diabetic patients (Miller and Richman 2016). Type 1 DM represents almost 10% of cases developed mainly in children or young adults where both genetic susceptibility and environmental triggers (viral, bacterial infection) cooperate in the progression of the disease. In Type 1 DM the auto-immune reaction destroys β-cells in the islets of the Langerhans of the pancreas thus β-cell replacement may be a promising therapeutic option (van Belle et al. 2011). Pancreas or pancreas + kidney clinical transplantation results in 81.5 or 89% 1 year survival, respectively, however the 10 year survival currently is around 75% only, thus further research is much needed to improve β-cell replacement in insulin dependent diabetes (Lysy et al. 2016).

Islet cells located in the tissue microenvironment in vivo are surrounded by a network of cell–cell and cell-extracellular matrix (ECM) connections. Dissected islets require the re-establishment of cell–matrix interactions in order to preserve hormone secretion and viability (Wang and Rosenberg 1999). Collagen, as an ECM component in connective tissues can support spheroid integrity and modulate cell signalling *e.*g. via collagen-integrin interactions (Riopel and Wang 2014). Encapsulation of rat embryonic pancreas precursors in collagen type I matrix incorporated in polyethylene glycol hydrogel (PEG) supported precursor cell development (Mason et al. 2009). An alternative to primary culture is to use immortalized mouse insulinoma as a model of β-cells where MIN6 cells are grown in rotation providing 250 µm spheroids (Tanaka et al. 2013). A previous study also described a three-dimensional co-culture system of islet and hepatocyte hybrid spheroids in concave microwell plates to grow and maintain islets in vitro (Jun et al. 2013).

In this manuscript we describe an advanced three-dimensional tissue culture technique. “*Real Architecture For 3D Tissue”* (RAFT™) combines the advantages of ECM collagen composition with three dimensional expansion. RAFT™ recently has been introduced in the research field of human ophthalmology as an ex vivo tissue culture system for culturing limbal fibroblast (Massie et al. 2015a), limbal epithelial cells (Massie et al. 2015b) and for improved interaction of corneal stem cells with limbal epithelial cells (Kureshi et al. 2015). Here we report, for the first time how RAFT™ tissue culture platform preserves pancreatic islet morphology, aids islet viability and supports insulin and glucagon production.

## Materials and methods

### Ethics statement

All mouse studies were done in accordance with national and international laws and regulations of animal experiments and were reviewed and approved by the Regional Animal Health Authorities, Pest County, Hungary, and by the Joint Local Ethics and Animal Welfare Comitee of Biotalentum Ltd. in possession of an ethical clearance 42/2015.

### Islet preparation and maintenance

Mice (CD-1 123^®^, Charles River, Freiburg, Germany) were sacrificed by cervical dislocation then disinfected by 70% ethanol (Molar Chemicals, Halásztelek, Hungary). Skin was removed and the abdominal cavity was opened. Pancreas was carefully dissected from surrounding tissues and transferred into a sterile plastic container (Corning Life Sciences, Corning, NY, USA) with Hanks Balanced Salt Solution (HBSS) without phenol red (Sigma-Aldrich). Fatty tissue was removed and the pancreas was cut into small pieces. Collagenase powder IX (2 mg/pancreas, Sigma-Aldrich, Budapest, Hungary) was dissolved in 1.5 ml HBSS. The pancreas-collagenase solution was manually shaken continuously for 12 min in a 37 °C water bath. The digestion was stopped by adding cold HBSS in excess. The suspension was centrifuged with 1000 rpm for 8 s at 4 °C. The pellet was suspended in HBSS and divided into two 100 mm Petri-dishes (Corning Life Sciences). The manual picking of islets was performed using 27G needles (Becton-Dickinson, Budapest, Hungary). The isolates were transferred using 200 µl pipette tip to a 60 mm Petri-dish containing HBSS on ice. After centrifugation (8 s, 1000 rpm, 4 °C) islets were resuspended in mouse embryonic stem cell (mESC) conditioned medium (MES) and maintained in 60 mm Petri-dishes (Corning Life Sciences) in a humidified incubator at 37 °C, 5% CO_2_ (Thermo Fisher Scientific, Budapest, Hungary). Media was replaced daily.

The islets were monitored under microscope equipped with 3D imaging module (AxioImager system with ApoTome, Carl Zeiss MicroImaging GmbH, Jena, Germany) controlled by AxioVision 4.8.1 Microscope software (Carl Zeiss MicroImaging GmbH). After 3 days islets were plated for subsequent analysis as described under each experimental setup. Medium was replaced on every third day.

### Preparation of MES medium

In order to harvest mouse embryonic stem cell (mESC) conditioned medium HM1 ESCs (129SV/Ola; kindly provided by Dr. Jim McWhir, Roslin Institute, Easter Bush, UK) (Selfridge et al. 1992) were cultured in T25 flasks (Corning Life Sciences) on mitomycin C (Sigma-Aldrich) treated fibroblast feeder cells in basic ESC medium (DMEM-High Glucose supplemented by 15% FCS, 1% (V/V) L-Glutamine, 1% (V/V) Non-Essential Amino Acids, 1% (V/V) Penicillin/Streptomycin, 0.2% (V/V) 2-Mercaptoethanol, all purchased from Thermo Fisher Scientific and 1/10,000 (V/V) Leukemia Inhibitory Factor, Merck Millipore, Budapest, Hungary), as previously described (Nagy et al. 1993). On every second day the supernatant was harvested from the cells, filtered through 0.2 µm filter and stored at 4 °C for 1 week. MES medium was prepared by diluting mESC conditioned medium and fresh basic ESC medium in 1:1 ratio.

### Preparation of RAFT™ cultures

After 3 days of maintenance, islets were diluted to plate an average 3 islets per well for subsequent analysis in 96-multiwell RAFT™ (3D) plates according to the instructions of the manufacturer (TAP Biosystems, Lonza, Cologne, Germany). Briefly, islets were dispensed in the chilled mixed collagen solution of the RAFT™ kit (containing 2.8 ml 10X MEM medium, 22.4 ml 2 mg/ml rat tail collagen type I, 1.624 ml neutralizing solution and 1.2 ml islets for 96-well tissue culture plate) according to the RAFT™ protocol. We plated 240 µl mixed collagen solution per well and incubated the plate at 37 °C to form a hydrogel for 15 min. Then we placed the RAFT™ absorbers on the top of the hydrogel in a laminar flow hood at room temperature (RT) for 15 min. After removing the absorbers we immediately loaded 200 µl MES medium per well. Medium was replaced on every third day.

### Viability staining

Islets were plated either in 96-well glass bottom tissue culture plates (2D) (Poly-D-Lysine coated, 5 mm glass diameter, MatTek Corp., Ashland, MA, USA) or in RAFT™ plates according to the instructions of the manufacturer (TAP Biosystems, Lonza) an average 3 islets per well in 200 μl medium. Suspension culture (SC) was maintained in sterile non-adherent 60 × 15 mm Petri dishes (Greiner Bio-One, Kremsmünster, Austria). Medium was replaced on every third day. At the indicated time points both 2D, RAFT™ and SC were PBS washed and 1 µM Calcein violet, AM was added (Molecular Probes, Thermo Fisher Scientific) diluted in PBS containing 1.2 mM CaCl_2_, 0.5 mM MgCl_2_ for 30 min at 37 °C. After gentle PBS washing islets were incubated with Annexin V Alexa Fluor^®^ 488 (Life Technologies, Budapest, Hungary, 2.5:100) in Annexin V binding buffer (0.01 M HEPES, 0.14 M NaCl and 2.5 mM CaCl_2_, Sigma-Aldrich) for 15 min in the dark at RT. Before the acquisition propidium-iodide (PI, 10 μg/ml, Sigma-Aldrich) was added in AnnexinV binding buffer to dilute AnnexinV Alexa Fluor^®^ 488 5X. At the end, RAFT™ disks or islets from SC were placed on a glass slide and covered by coverslip (Menzel-Gläser, Braunschweig, Germany). Islets were analyzed immediately on Olympus Fluoview FV1000 confocal laser scanning microscope (Olympus Life Science Europa GmbH, Hamburg, Germany). Pictures were generated by FV-ASW 4.0 Viewer software (Olympus Life Science Europa GmbH). Representative images are shown of 3 replicates (n = 3) of each culture method.

### Immunofluorescent staining

In order to validate the isolates anti-insulin and anti-glucagon immunohistochemistry was performed immediately after isolation. Islets were fixed in 4% (V/V) PFA for 20 min at RT, permeabilized with 0.1% (V/V) Triton X-100 for 5 min (Sigma-Aldrich) and blocked in 3% (M/V) bovine serum albumin (BSA, Sigma-Aldrich) for 1 h at RT. The islets were incubated with primary antibodies for overnight at 4 °C (anti-glucagon 1:50 (N–17) and anti-insulin 1:100 (H–86), Santa Cruz Biotechnology, Santa Cruz, CA, USA) diluted in the blocking buffer. After PBS washing hormone production was visualized by secondary antibodies: anti-rabbit Alexa Fluor^®^ 488, anti-goat Alexa Fluor^®^ 488 (1:2000, Thermo Fisher Scientific) diluted in the blocking buffer. After PBS washing 0.2 µg/ml 4, 6-diamidino-2-phenylindole (DAPI, Sigma-Aldrich) was used for nuclei counterstaining for 20 min at RT. The islets were observed under fluorescent microscope equipped with 3D imaging module, as described above.

For time-course experiment islets were plated either in 96-well glass bottom tissue culture plates (2D) (Poly-D-Lysine coated, 5 mm glass diameter, MatTek Corp.) or in RAFT™ plates according to the instructions of the manufacturer (TAP Biosystems, Lonza) an average 3 islets per well in 200 μl medium. Suspension culture was maintained in sterile non-adherent 60 × 15 mm Petri dishes (Greiner Bio-One). Medium was replaced on every third day. At the indicated time points both 2D, RAFT™ and SC were PBS washed (rinsed 3 times for 5 min) then fixed by 100 µl 3.7% (V/V) PBS buffered formaldehyde for 10 min at RT. The formaldehyde solution was replaced with 100 µl quenching solution (1/50 dilution of stock solution (50 mM Tris–HCL pH 7.5 + 1 M Glycine) in PBS), then islets were PBS washed. Islets were permeabilized by either 100 µl 0.1% Triton X-100 (2D, SC) or 1% Triton X-100 (RAFT™) solution for 4 min. After PBS washing primary antibodies were added: anti-glucagon 1:50 (N-17) and anti-insulin 1:100 (H-86) (Santa Cruz Biotechnology) diluted either in 1% BSA (2D, SC) or in 1% BSA 0.2% Triton X-100 (RAFT™) for overnight at 4 °C. After PBS washing secondary antibodies: anti-rabbit Alexa Fluor^®^ 488, anti-goat Alexa Fluor^®^ 594 (1:2000, Thermo Fisher Scientific) were diluted and added either in 1% BSA (2D, SC) or in 1% BSA 0.2% Triton X-100 (RAFT™) and incubated for 2 h at RT in the dark. After PBS washing RAFT™ disks and islets from SC were place on glass slide and covered by coverslip (Menzel-Gläser). Islets were analyzed on Olympus Fluoview FV1000 confocal laser scanning microscope (Olympus Life Science Europa GmbH, Hamburg, Germany). Pictures were generated by FV-ASW 4.0 Viewer software (Olympus Life Science Europa GmbH). Representative images are shown of 3 replicates (n = 3) of each culture method.

### Nucleic acid isolation

Islets were plated either in 96-well TC-treated tissue culture plates (2D) (Corning Life Sciences) or in RAFT™ (3D) plates according to the instructions of the manufacturer (TAP Biosystems, Lonza) an average 3 islets per well in 200 μl medium. Suspension culture was maintained in sterile non-adherent 60 × 15 mm Petri dishes (Greiner Bio-One). Medium was replaced on every third day. At the indicated time points both 2D, RAFT™ and SC cultures were PBS washed. 2D cultures were trypsinized (Lonza); RAFT™ were pooled and digested by 1 mg/ml collagenase IV (Sigma-Aldrich) for 30 min at 37 °C, manually shaken in serum free DMEM (Gibco). Subsequently islets were pelleted (1 min, 2000 rpm, Eppendorf) and PBS washed. Total RNA was purified as described previously (Fabian et al. 2011); columns and washing buffer were purchased from Bioneer (Viral RNA Extraction Kit)(Hørsholm, Denmark). Briefly, cells were washed twice with PBS, incubated in lysis buffer (RA1; Macherey–Nagel), with 1% 2-Mercaptoethanol (Sigma-Aldrich), then harvested and mixed with 70% ethanol (Molar Chemicals). The mixture was transferred through columns (Bioneer) and washed with 350 µl 80% ethanol. Then 95 μl DNase reaction mixture (Macherey–Nagel, Budapest, Hungary) was loaded onto the columns and they were incubated at room temperature (RT) for 15 min. After the DNase digestion, the columns were washed with 150 μl mixture (1:1) of RA1 lysis buffer (Macherey–Nagel) and ethanol, subsequently washed with 600 μl and 300 μl W2 washing buffer. Total RNA was eluted in 30 µl RNase free-water. One µl RNase inhibitor (20 U/µl Applied Biosystems, Foster City, CA, USA) was added to the samples. The quality and quantity of the isolated RNA were measured with NanoDrop1000 Version 3.8.1. (Thermo Fisher Scientific).

### Gene expression analysis

Reverse transcription from 1 µg of total RNA was performed with the High-Capacity cDNA Archive Kit (Applied Biosystems) in a total volume of 10 µl according to the manufacturer’s protocol. After dilution with 15 μl of ultrapure water (Applied Biosystems) cDNA was used as template for gene expression analysis. Quantitative real-time PCR (qRT-PCR) was performed on the LightCycler^®^ Nano Instrument (Roche) using gene-specific primers with SYBR Green protocol as described previously (Kata et al. 2016). Briefly, for cycling each 10 μl PCR reaction contained 20 ng cDNA, 250 nM primers and 5 μl FastStart Essential DNA Green Master (2X, Roche). Primers were designed using the online Roche Universal Probe Library Assay Design Center. The quality of the primers was verified by MS analysis provided by Bioneer. The PCR primer sequences are presented in Online Resource 1. The PCR protocol was as follows: enzyme activation at 95 °C for 10 min, 50 cycles of denaturation at 95 °C for 15 s, annealing and extension at 60 °C for 30 s. All PCRs were performed with 5 replicates. After amplification, melting curve was checked to verify the specificity of the PCR reactions. Three control genes: glyceraldehyde-3-phosphate dehydrogenase (*Gapdh*), beta-actin (*Actb*) and hypoxanthine phosphoribosyltransferase (*Hprt*), were used to normalize mRNA levels between different samples (Puskas et al. 2005). We obtained the same tendency in gene expression changes (data not shown) when we normalized to these housekeeping genes separately. The presented relative gene expression ratios were normalized to *Gapdh* gene, calculated using the comparative CT method (2^−ΔΔCT^). Fold change refers to 2^−ΔΔCt^. Fold change values presented here are gene expression values on day 18 of islet culture in a given setup compared to day 1 of SC. All values are presented as mean  ±  SD.

### Statistical analysis

Statistical analysis was performed using two-tailed, homoscedastic Student’s *t* test to evaluate the statistical significance (set at * *p* < 0.05, ** *p* < 0.01, *** *p* < 0.001) between two given experimental groups (samples at 18th day were pair-wise compared: SC to RAFT™, 2D to RAFT™ and 2D to SC).

## Results and discussion

### Validation of the islets

**Fig. 1:**
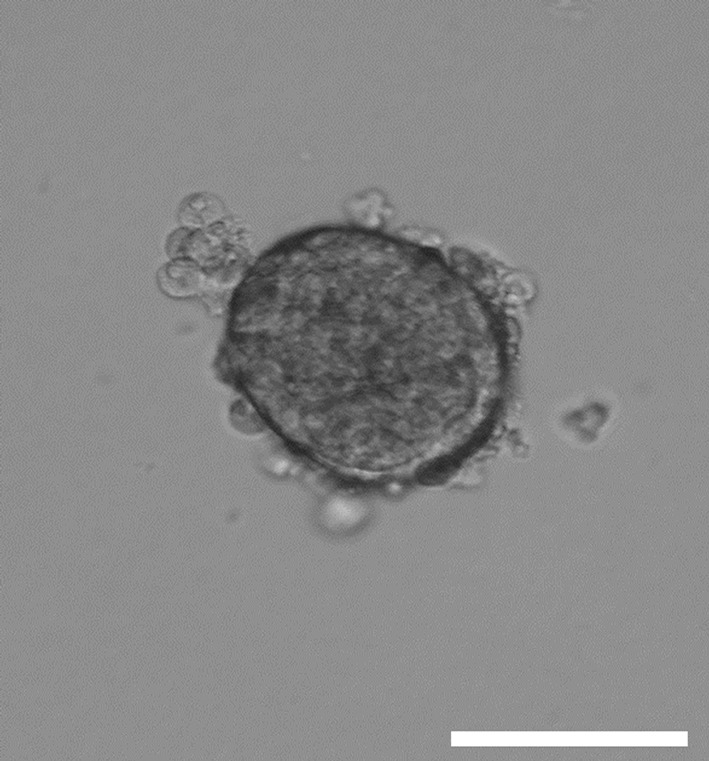
Representative morphology of mouse islets upon isolation. *Scale bar* indicates 100 µm

**Fig. 2:**
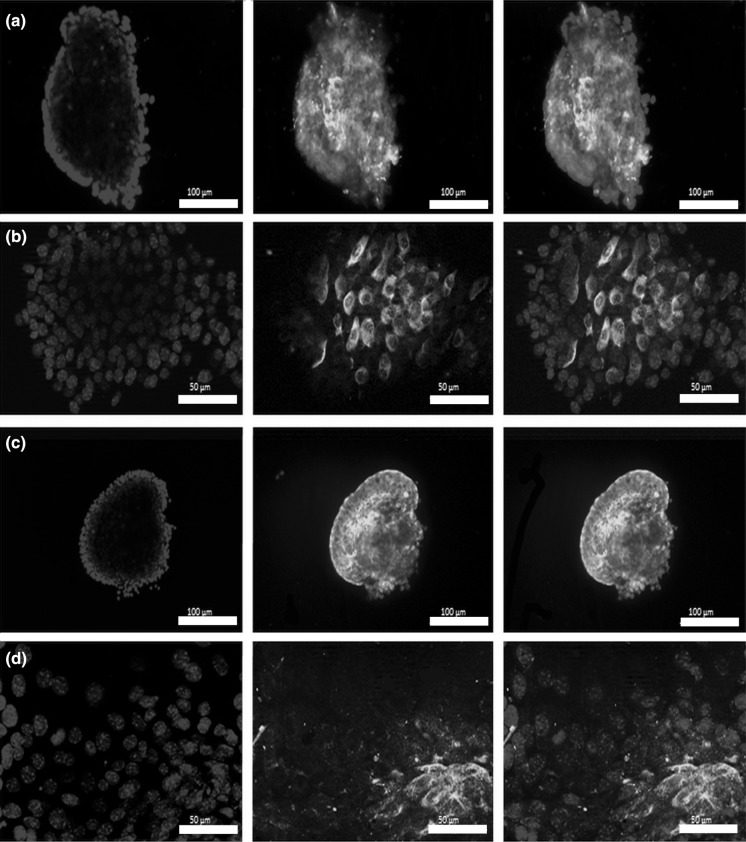
Validation of mouse pancreatic islets right after isolation by immunofluorescent (IF) staining. We performed glucagon (**a** and **b**, *green*) and insulin (**c** and **d**, *green*) IF staining counterstained with DAPI to visualize nuclei (*blue*). *Scale bars* indicate 100 µm (**a** and **c**), 50 µm (**b** and **d**). (Color figure online)

Islet morphology was assessed upon isolation. Using the collagen digestion method we were able to isolate intact pancreatic islet spheroids as it is demonstrated by light microscopy picture (Fig. 1). In order to further validate the pancreatic islets, glucagon (Fig. 2a, b) and insulin (Fig. 2c, d) immunofluorescent staining was performed right after isolation. DAPI nuclei staining contoured the spheroid morphology of the islets (Fig. 2). In line with the literature, mouse pancreatic islets consisted of more insulin producing β-cells (Fig. 2c) than glucagon producing a-cells (Fig. 2a) (Dolensek et al. 2015). Insulin staining showed the characteristic insulin core (Suckale and Solimena 2008) of murine islets (Fig. 2c).

### RAFT™ preserves spheroid morphology and improves viability of the islets

**Fig. 3:**
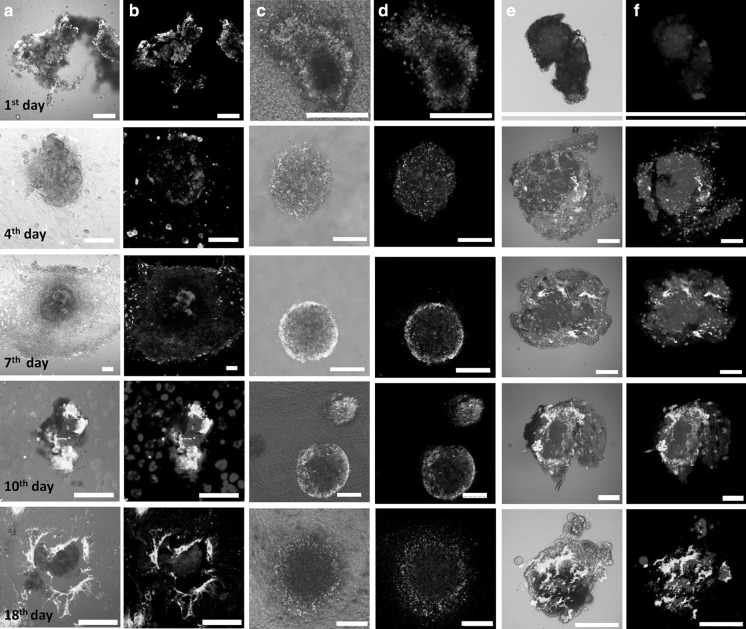
Viability staining and morphology of the islets in different culture conditions after the indicated time points detected by confocal laser scanning microscopy. (**a**, **b** columns) monolayer cultures, (**c**, **d** columns) RAFT™ cultures, (**e**, **f** columns) suspension cultures. In order to visualize the integrity of the islets bright field images (**a**, **c**, **e** columns) were merged with the corresponding fluorescent images (**b**, **d**, **f** columns). Live cells were visualized by Calcein violet AM (*blue*), apoptotic and dead cells were visualized by Annexin V Alexa Fluor^®^ 488 (*green*) and propidium-iodide (*red*) staining, (n = 3). All *scale bars* indicate 100 µm. (Color figure online)

We followed islet morphology in different culture conditions throughout the 18 day culture period by confocal laser scanning microscopy. As control, islets cultured in monolayers (Fig. 3a, b columns) or in suspension (Fig. 3e, f columns) were compared to RAFT™ cultures (Fig. 3c, d columns). In order to visualize the integrity of the islets bright field images (Fig. 3a, c, e columns) were merged with the corresponding fluorescent images (Fig. 3b, d, f columns).

At the same time islet viability was also monitored. Cells were visualized by the live-cell dye Calcein violet AM (blue), early-apoptotic and dead cells were visualized by Annexin V Alexa Fluor^®^ 488 (green) and propidium-iodide (red) staining. Annexin V was applied to detect phosphatidyl-serine exposure of apoptotic cells and PI to detect late apoptotic and necrotic death (Bratosin et al. 2005; Palma et al. 2008). Between days 4 and 7, gradual morphological changes were noticed with the outspreading of fibroblast-like cells in the monolayer cultures (Fig. 3a, b columns), whereas islets inoculated directly within the collagen gel (RAFT™) preserved their globular shape (Fig. 3c, d columns). Between day 10 and 18 the continued outgrowth of fibroblast-like cells resulted in the loss of islet integrity in monolayer cultures (Fig. 3a, b) whereas RAFT™ maintained spheroid structures (Fig. 3c, d). These results are significant in the light of previous work showing that maintenance of islet morphology is critical to improve graft function and revascularization after transplantation (Ravi et al. 2015). The intensity of Calcein violet AM corresponding to intracellular esterase activity proportional to cell viability was much higher, consistent and long-lasting in RAFT™ type I collagen embedded islet cells compared to 2D cultures. Moreover, Calcein violet AM signal mainly localized in the viable core of the islets indicating a satisfactory nutrient and oxygen supply (Fig. 3c, d). In the monolayer cultures mainly fibroblast-like cells produced Calcein violet AM signal since the islets were disintegrated and overgrown by stromal fibroblast-like cells by the 7th day of culture (Fig. 3a, b).

Islets maintained in the standard suspension culture preserved their spheroid morphology although they showed propidium-iodide positive necrotic areas after the 7th day of culture (Fig. 3e, f). Islets were mostly damaged and necrotic, confirmed by massive propidium-iodide staining by the 4th day in 2D samples (Fig. 3a, b) whereas islets in RAFT™ showed scattered and low intensity Annexin V staining only at the periphery of the spheroid during the entire 18 day culture period.

### RAFT™ system maintains insulin expression

**Fig. 4:**
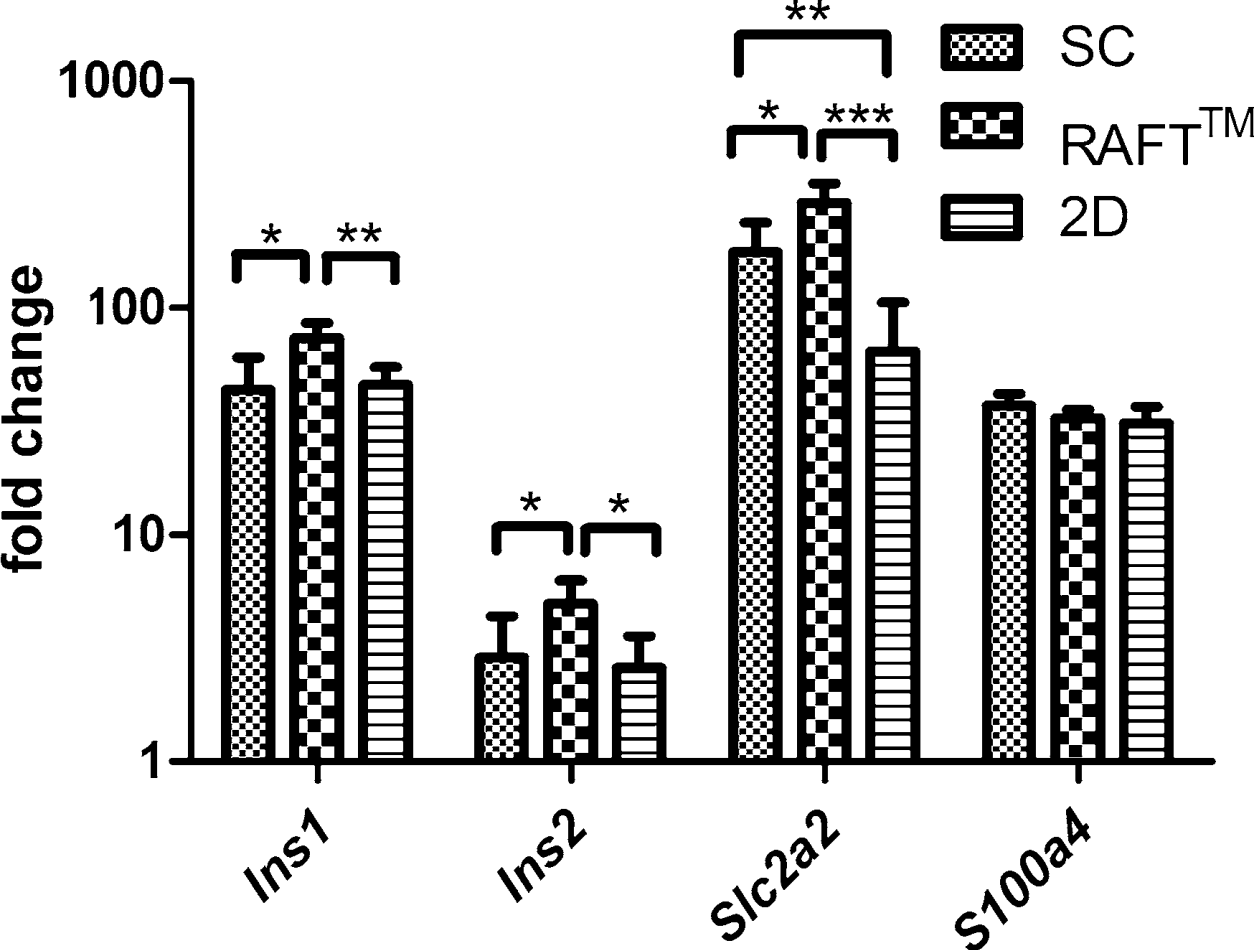
QRT-PCR analysis of gene expression in suspension (SC), RAFT™ and monolayer (2D) cultures on day 18 of culture. Expression of these selected genes was compared both in the case of SC, RAFT™ and 2D to the corresponding gene expression on day 1 of the standard SC. Fold change values were calculated as described in Materials and methods, (n = 5). Statistical significance was set at *p < 0.05, **p < 0.01, *** *p* < 0.001. (Color figure online)

**Fig. 5:**
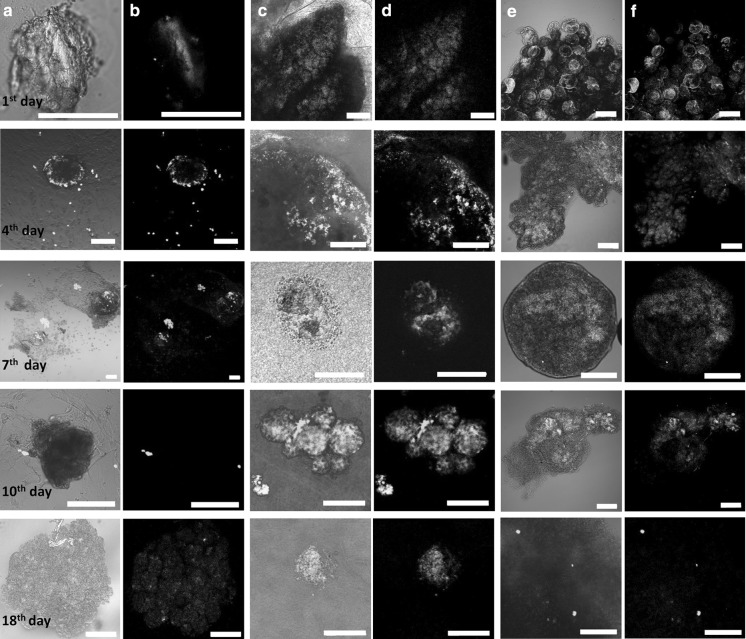
Evaluation of insulin-producing β-cells and glucagon-producing α-cells by immunofluorescent (IF) staining in different culture conditions after the indicated time points detected by confocal laser scanning microscopy. (**a**, **b** columns) monolayer cultures, (**c**, **d** columns) RAFT™ cultures, (**e**, **f** columns) suspension cultures. In order to visualize the integrity of the islets bright field images (**a**, **c**, **e** columns) were merged with the corresponding fluorescent images (**b**, **d**, **f** columns). Insulin production was visualized by anti-rabbit Alexa Fluor^®^ 488 (*green*) and glucagon by anti-goat Alexa Fluor^®^ 594 (*red*), (n = 3). All *scale bars* indicate 100 µm. (Color figure online)

Quantitative real-time PCR (qRT-PCR) analysis was carried out to investigate the expression of islet specific genes (insulin-1, *Ins*-*1*; insulin-2, *Ins*-*2*; glucagon, *Gcg*; glucose transpoter-2, *Slc2a2*) and fibroblast specific protein-1 (*S100a4*). Primers used in this study are summarized in Online Resource 1. Expression of these selected genes was compared both in the case of SC, RAFT™ and 2D to the corresponding gene expression of SC on day 1. *Ins*-*1* significantly increased in RAFT™ pair-wise compared to SC (* *p* < 0.05) or 2D (** *p* < 0.01). *Ins*-*2* also showed elevated expression in RAFT™ pair-wise compared to SC (* *p* < 0.05) or 2D (* *p* < 0.05) (Fig. 4), respectively. Although immunofluorescent staining of glucagon showed robust signal intensity in RAFT™ until the 10th day (Fig. 5c, d), *Gcg* gene expression was almost undetectable until day 18 in both culture conditions. The glucose sensor of pancreas, the gene of Glut-2 glucose transporter (Efrat 1997), *Slc2a2* showed the highest expression in RAFT™ culture system reinforcing the concept that the three dimensional collagen embedded growth favors islet functionality and survival (Fig. 4). Since the expression of *S100a4,* a fibroblast marker was similar among different culture conditions further studies are needed to ascertain the type of fibroblast-like stromal cells spreading out of the disintegrated islets in the 2D system (Fig. 3a, b).

Indirect immunofluorescent staining was used to validate qRT-PCR data and to assess insulin and glucagon production during culturing. Bright field images were merged with the corresponding fluorescent images in order to visualize the integrity of the islets. While islets were disintegrated in the monolayer cultures at day 7 with diminished insulin and glucagon expression (Fig. 5a, b), insulin and glucagon production showed intense signal and it was sustained continuously not only in the standard SC but also in RAFT™ for 18 days (Fig. 5c, d). RAFT™ cultures showed the brightest hormone staining over the 18 day culture period. This result is superior to previously published results with a peptide amphiphile nanostructured gel-like scaffold which maintained islet viability and hormone production for 14 days only (Lim et al. 2011).

Type I collagen belongs to a group of natural 3D scaffolds which resembles in vivo conditions resulting in enhanced cell survival and functionality (Ravi et al. 2015). In RAFT™ cultures, whether the collagen matrix serves as a 3D scaffold substituting the basement membrane or stimulates tissue-specific genes via cell surface, e.g. integrin receptors have to be further investigated. Traditional monolayer cultures served to understand basic cellular physiology but 3D models enable multiple cell-cell-ECM interactions to investigate how cell-cell and cell-ECM signals converge on a particular cell type. Our data showed that the RAFT™ system constitutes a proper 3D collagen matrix for pancreatic islets mirroring the structure of the in vivo microenvironment. Although type I collagen has been described previously for islet maintenance ex vivo (Mason et al. 2009; Riopel and Wang 2014) RAFT™ provides an advanced standardized system. Additionally to the described benefits, the RAFT™ system using embedded cells provides a robust technology that is applicable to automation for semi-high throughput or high throughput assays in pharmacological applications.

## Conclusions

To the best of our knowledge this is the first report about RAFT™ tissue culture system in the application of pancreatic islet maintenance ex vivo. We have investigated the viability and architecture of islets in RAFT™ supplemented by the characterization of α-, β-cell functional activity. Overall, the use of RAFT™ provided excellent results in preserving islet spheroid viability, structure integrity and insulin, glucagon production for at least 18 days ex vivo. Based on our data RAFT™ can be a promising tool both in basic and applied research to further optimize current research models and therapies in the field of diabetes.

## Electronic supplementary material

Below is the link to the electronic supplementary material.

Supplementary material 1 (XLSX 10 kb)
